# Human αB-crystallin as fusion protein and molecular chaperone increases the expression and folding efficiency of recombinant insulin

**DOI:** 10.1371/journal.pone.0206169

**Published:** 2018-10-19

**Authors:** Mohsen Akbarian, Reza Yousefi

**Affiliations:** Protein Chemistry Laboratory, Department of Biology, College of Sciences, Shiraz University, Shiraz, Iran; University of Colorado Denver School of Medicine, UNITED STATES

## Abstract

Low expression and instability are significant challenges in the recombinant production of therapeutic peptides. The current study introduces a novel expression and purification system for human insulin production using the molecular chaperone αB-crystallin (αB-Cry) as a fusion partner protein. Insulin is composed of A- and B-chain containing three disulfide bonds (one intarchain and two interchains). We have constructed two plasmids harboring the A- or B-chain of insulin joined with human αB-Cry. This system is suitable for cloning of the genes and for directing the synthesis of large amounts of the fusion proteins αB-Cry/A-chain (αB-AC) and αB-Cry/B-chain (αB-BC). The construction of vectors, their efficient expression in *Escherichia coli* and simple purification of the fusion proteins and two insulin chains are described. A large amount of the recombinant fusion proteins with high purity was obtained by applying a single step anion exchange chromatography or metal chelate affinity. The insulin A- and B-chain were released from the fusion proteins using cyanogen bromide cleavage. The insulin peptides were obtained with an appreciable yield and high purity using one-step gel filtration chromatography. To increase efficiency of chain combination to produce insulin, αB-Cry was used under oxidative conditions. The purification of natively folded insulin was performed by phenyl sepharose hydrophobic interaction chromatography. Finally, using an insulin tolerance test in mice and various biophysical methods, the structure and function of purified human recombinant insulin was compared with authentic insulin, to verify folding of insulin to its native state. Overall, the novel expression system using αB-Cry is highly demanding for producing human insulin and functional protein. The procedure for αB-Cry-mediated insulin folding could be also applicable for the large-scale production of this highly important therapeutic peptide hormone.

## Introduction

Changes in diet and lifestyle are two important factors causing the worldwide dramatic increase in the incidence of diabetes [1]. Currently, about 0.7% of the world population suffer from insulin-dependent diabetes mellitus [2]. Both type I and type II diabetic patients require insulin, but because of developing insulin resistance, the late stage type II diabetes patients use larger amounts of this hormone [3, 4]. Due to the cumulative incidence of this metabolic disease among children and adults, insulin is now at the top of the list of therapeutic peptides in high demand [5]. Insulin controls the storage and use of sugars and if by its dysfunction, the blood sugar homeostasis is not well preserved for long periods, serious complications will appear in different tissues [6].

Methods for producing insulin initially used extraction of the native hormone from the animal pancreas [7]. However, the use of bovine or pig insulin is associated with the appearance of significant adverse effects (e.g. allergy) [8]. Due to its reputable production efficiency, recombinant DNA technology is now used for human insulin production. *Escherichia coli* and *Saccharomyces cerevisiae* are the most important hosts for production of human insulin [9]. Because of limitation in production capacity and high production cost of insulin, current manufacturing technologies are not able to meet the human demand [10]. In spite of the several attempts which have been recently made to express proinsulin and insulin in bacteria, the direct production of (pro) insulin in the bacterial host is generally difficult. Insulin is composed of two chains, A and B, that are joining by two disulfide bonds with a third disulfide bond within the A-chain. Due to their small size, insulin peptide chains are very prone to be degraded by the host’s proteases [11]. To overcome this challenge, an approach for a successful expression and purification system is to design recombinant insulin chains fused to an appropriate partner protein. Subsequently, the target insulin peptides are released from the fusion protein by either chemical or enzymatic cleavage at the corresponding site of carrier-peptide junction. Choosing an appropriate fusion protein partner is very important because according to previous studies, certain fusion protein partners could greatly improve the stability and the level of expression of the target proteins [12–14]. In the pancreatic tissue, a single chain proinsulin molecule is produced with the A-chain (21 amino acids) and B-chain (30 amino acids) linked together by the C-peptide (31 amino acids). The formation of native insulin from proinsulin requires a two-step process i.e. formation of the characteristic pattern of disulfide bridges which is followed by proteolytic cleavage and subsequent release of the C-peptide [15].

Recently β-galactosidase (β-gal) has been used as a fusion protein partner, with plasmids containing the DNA sequence encoding the A- or B-chain of human insulin. The production of the fusion protein is intracellular and it appears as cytoplasmic inclusion bodies in the bacterial host (*Escherichia coli*) [16]. However, the large size of β-gal fusion protein partner (1000 amino acids) limits the yield of insulin to a significant level. Moreover, during translation, the fusion proteins (β-gal and insulin A- or B-chain) became detached from the ribosome resulting in premature chain termination [17, 18]. Also, relatively high concentrations of the A- and B-chain are required for the efficient folding during insulin chain combination to produce functional insulin. The large amounts of insulin chains at this step usually result in peptide chain aggregation which significantly reduces the yield of natively folded insulin [19, 20]. The big demand for affordable human insulin therefore requires the development of a more efficient production system and also introduction of suitable folding conditions. To overcome the above mentioned complications, in our system for expression, purification and chain combination, we have used human αB-crystallin (αB-Cry) as both fusion protein partner and a molecular chaperone mediating insulin folding. The intrinsic ability of this chaperone protein to form large oligomers [21] may also facilitate the purification of the target peptides. Overall, we have designed a streamlined expression and purification approach for the insulin A- and B-chain which each step is straightforward with the high yields. Our method might be also applicable to the production of other therapeutic peptides.

## Materials and methods

Bis-1-anilino-8-naphthalene sulfonate (bis-ANS), thioflavin T (ThT), sodium tetrathionate, sodium sulfide, cyanogen bromide, and other chemicals were purchased from Sigma. Standard insulin and goat anti-rabbit IgG peroxidase were purchased from Sigma-Aldrich Company. The dialysis tube (2 kDa cut-off) was purchased from Spectrum Scientific Company. Anti αB-Cry antibody was a generous gift of Professor Samuel Zigler (Johns Hopkins School of Medicine). Gel filtration media and Ni-NTA matrix were from GE Healthcare and Qiagen, respectively.

### Gene and plasmid construction

**Fig 1:**
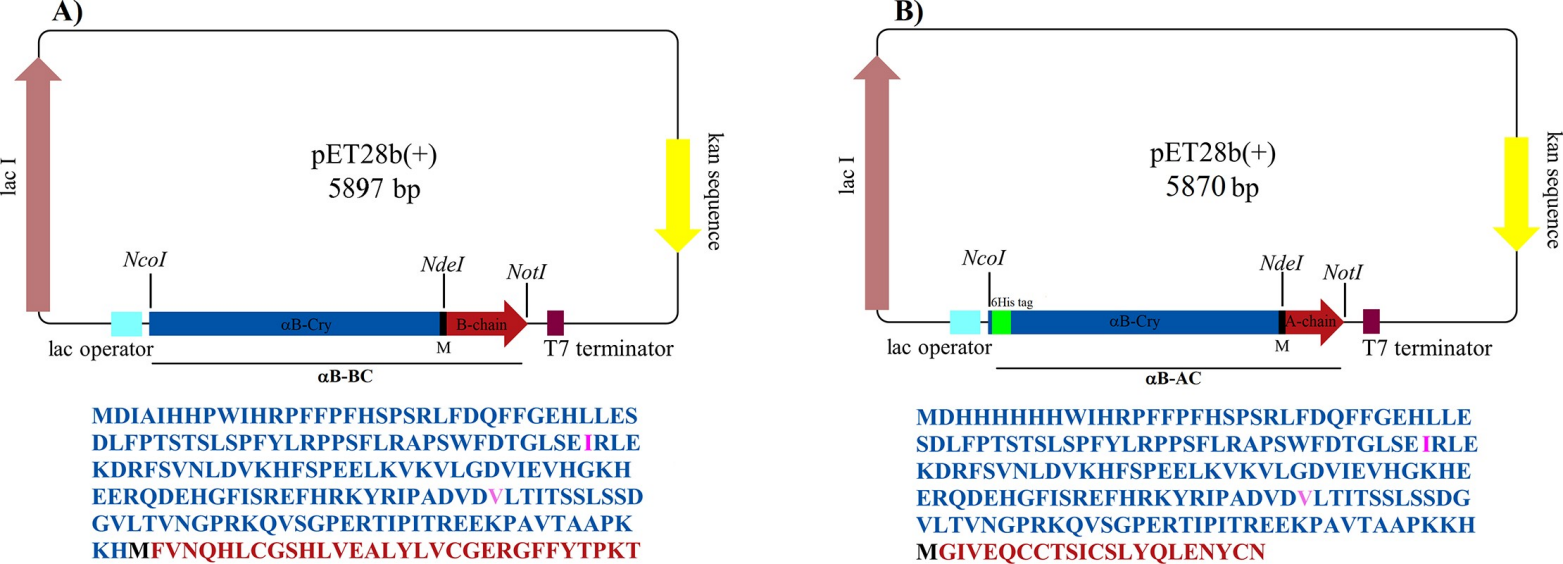
Schematic illustration of sequence of the designated vectors. pET28b (+) vectors harboring fusion genes are depicted here. The positions of *Nco*I, *Nde*I and *Not*I restriction sites are also indicated. (**A**) and (**B**) respectively stand for the vectors including the fusion gene of αB-BC and αB-AC. The translation frame under the vectors correspond to the amino acid sequence of the fusion proteins. The sequence of αB-Cry fusion partner is indicated in blue. (**M**) stands for the single letter abbreviation of methionine serving as the CNBr cleavage site. The amino acid sequence of B- and A-chain of human insulin is indicated in red. Isoleucine and valine residues (in pink) generated by site direct mutagenesis in αB-Cry gene instead of methionine and proline, respectively.

A schematic representation of the expression vector pET28b (+) (Novagene) and additional sequence information of the construct are given in **Fig 1**. As shown in **Fig 1A** and **1B**, the expression constructs consist of human αB-Cry (*CRYAB*) ligated to the B-chain (αB-BC) or the A-chain (αB-AC) of the human insulin genes. The fusion gene is flanked by *Nco*I and *Not*I restriction sites. Also, the *Nde*I site was designed to encode a methionine residue which provides a CNBr cleavage site at the end of the fusion partner protein (αB-Cry). Additionally, in the sequence of αB-Cry, methionine 68 and proline 130 were replaced, respectively, with isoleucine and valine to provide resistance against cleavage by CNBr and formic acid. These substitutions were made using QuikChange II XL Site-Directed Mutagenesis Kit (Stratagene) following the manufacturer's instruction. To produce the constructs, the fusion gene sequences with designated restriction sites were chemically synthesized and then cloned into pET28b (+). The amino acid sequence of the desired fusion genes is presented in **Fig 1**. The pET28b (+) containing the A-chain was engineered by designing a 6 His-tag at the N-terminus of the *CRYAB* gene. This affinity tag was incorporated to facilitate the purification of the A-chain.

### Expression of the fusion proteins and isolation of inclusion bodies

The constructs were transformed into *E*. *coli* XL1-blue [22]. After extraction of the vectors (Miniprep Kit), the correct DNA sequence was confirmed by sequencing. Then, the constructs (αB-AC and αB-BC) with correct sequences were transformed into *E*. *coli* BL21 (DE3) (Stratagene). Typically, a single colony was used to inoculate a 15 mL overnight culture grown at 37°C in LB medium containing antibiotic (50 μg/mL kanamycin) with constant shaking. The overnight cultures were then used to inoculate 1000 ml Luria-Bertani (LB) medium supplemented with antibiotics. The cells were grown at 37°C to an optical density of 1 at 600 nm, prior to induction of protein expression by the addition of isopropyl-β-D-thiogalactopyranoside (IPTG) at a final concentration of 0.25 mM. The cells were grown overnight, then centrifuged at 7000 × g for 7 min and stored at -20°C, prior to further processing. To constitute an effective expression system for the peptides, we took advantage of the fusion partner αB-Cry, which promotes the formation of inclusion bodies in *E*. *coli*. These misfolded and insoluble aggregates offer multiple benefits such as enhanced protein expression (>30%), high purity, resistance to proteolytic digestion, and convenient isolation of the inclusion bodies [18, 23, 24]. For partial purification and complete solubilization of the inclusion bodies, the cell pellets (1 gr) containing αB-AC and αB-BC fusion proteins, were resuspended separately in 10 mL of lysis buffer (50 mM Tris-HCl, pH 8.0, 5 mM EDTA) and then homogenized. The bacterial cells were lysed by sonication, and the cell debris was centrifuged by a low-speed centrifugation (6000 × g for 7 min). The pellets were further washed as a suspension in a wash buffer A (50 mM Tris-HCl, pH 8.0, 5 mM EDTA) with 0.1% (v/v) Triton X-100 and 1 M urea, sonicated for 30 seconds in an ice bath, and centrifuged (8000 × g, 4°C, 20 min). To collect the inclusion bodies (αB-AC and αB-BC), the debris was resuspended in 0.1gr/mL in the binding buffer B (20 mM Tris-HCl, pH 6.7, 5 mM 2-Mercaptoethanol) for αB-AC and binding buffer C (50 mMTris-HCl, pH 8.4, 5 mM 2-Mercaptoethanol) for αB-BC, both containing 8 M urea. The inclusion bodies containing the fusion proteins were also recovered from the debris by centrifugation at 10000 × g for 30 min at 4°C and the supernatants were passed through a filter (0.2 μM pore diameter) prior to the purification. In each step, purity of the protein samples was analyzed by 12% SDS–PAGE gel. Also, a 10 L of the culture medium was used for each fusion protein.

### Purification of the fusion proteins αB-AC and αB-BC

Purification of αB-AC and αB-BC fusion proteins were done using Ni-NTA (13 mL) (Qiagen, Germany) and DEAE Sepharose (Pharmacia) columns, respectively. The solubilized inclusion body containing αB-AC under reducing conditions (5 mM β-mercaptoethanol) was applied directly to a Ni-NTA (13 mL) column pre-equilibrated with binding buffer B (20 mM Tris-HCl, pH 6.7, 5 mM β-mercaptoethanol) containing 8 M urea. The column was washed with the respective binding buffer and the bound protein was eluted with elution buffer (20 mM Tris-HCl, pH 6.7, 5 mM β-mercaptoethanol) containing 8 M urea and 100 mM imidazole at a 1 mL/min flow rate. The eluted fractions were collected and analyzed on reducing SDS–PAGE and western blot. For purification of αB-BC, the solubilized inclusion body containing αB-BC was loaded on DEAE Sepharose column, pre-equilibrated in binding buffer C containing 8 M urea, at about 30 mg of total protein per mL of resin. The protein fraction of αB-BC was washed with equilibration buffer at pH 8.0 and the bound material was eluted with 100 mM NaCl in the same buffer.

### Gel electrophoresis and western blot analysis

Expression, isolation of the inclusion body and quality of purification were determined by visualization of total proteins, following separation by a 12% polyacrylamide gel electrophoresis. The protein bands were visualized using Coomassie brilliant blue (CBB) staining [25]. Also, cleavage of the fusion proteins and isolation of the peptides were analyzed on an 18% SDS-PAGE gel. αB-AC and αB-BC were identified by western blot analysis using a primary antibody which specifically recognizes the αB-Cry, and addition of the horseradish peroxidase second antibody (goat anti-rabbit IgG peroxidase). The visualization was achieved with diaminobenzidine as the enzyme substrate [26].

### Cleavage of the fusion proteins and purification of insulin A- and B-chain

The peptides (A- and B-chain of insulin) were cleaved from the fusion protein partner (αB-Cry) using CNBr. There are various chemical cleavage methods that offer an inexpensive procedure for removal of the fusion tags or partners. In this case, the purified and lyophilized αB-AC and αB-BC proteins which obtained from a 10 L of Luria broth (LB) culture medium were resuspended individually in 70% (v/v) formic acid at a concentration of 50 mg/mL. Also, CNBr (350 mg/mL) prepared in 70% (v/v) formic acid was added at a 100-fold molar excess over protein and the mixture was allowed to react for 24 h, at room temperature. The CNBr and formic acid were removed by overnight dialyzing against 5 L of double distilled water using an appropriate dialysis tube (2 kDa molecular weight cut-off). The precipitates containing αB-AC (~ 1300 mg) and αB-BC (~ 1700 mg) were stored at -20°C.

### The sulfitolysis experiment

The digested and lyophilized αB-AC and αB-BC were individually dissolved (100 mg/mL) in 300 mM Tris-HCl, pH 8.6 containing 8 M urea. S-sulfonated derivatives of the peptide mixtures were prepared by adding sodium sulfite and sodium tetrathionate to the final concentration of 250 and 80 mM, respectively. After incubation at room temperature for 3 h, the pH was adjusted to 5.0 with acetic acid [27] and the mixtures were dialyzed twice against 3 L of double distilled water. The resulting white precipitates containing ~1210 mg αB-AC and ~1630 mg αB-BC were centrifuged at 9000 × g for 45 min at 4°C and then lyophilized.

### Purification of the insulin peptide chains

The B-chain was separated from its fusion partner protein using gel filtration chromatography on a Sephadex G-50 (90 × 1, GE Healthcare) column which had been equilibrated in 1M glacial acetic acid and eluted with the same solvent [27, 28]. Due to the insolubility of the A-chain in 1M acetic acid, 20 mM Tris-HCl (pH 8.0) containing 8 M urea was used for purification of this peptide [29]. For purification of B-chain, the lyophilized S-Sulfonated derivatives (100 mg) were dissolved in 2 mL acetic acid (1M) and then loaded onto a Sephadex G-50 column. The column was equilibrated and the B-chain was eluted with 1M acetic acid. The flow rate and fraction size were 0.2 mL/min and 2 mL, respectively. The optical density of the fractions was determined at 276 nm. The A-chain was purified in the same way in 20 mM Tris-HCl pH 8.0 containing 8 M urea as the elution solvent. For gel filtration chromatography, all the buffers and solvents were degassed.

### Application of αB-Cry to increase the yield of insulin A-B chain combination

The combination of A- and B-chain was performed according to a standard protocol [20, 27]. The A- and B-chain (weight ratio of 2:1) were dissolved in degassed buffer (0.1 MGly/NaOH pH 10.5). Dithiothreitol was rapidly added to the peptide solution to a molar ratio 1:1 of SH to SSO_3_^2^. At the indicated time points (0, 12, 24, 36 and 48 h), the aliquots were withdrawn and precipitated proteins were removed by centrifugation (9000 × g for 30 min at 4°C). Refolding was performed under similar conditions in the presence of αB-Cry in a molar ratio of 0.04 for αB-Cry and insulin B-chain. The retention time of αB-Cry was determined on a C18 RP-HPLC column (S1 Fig). The yield of insulin A-B chain combination was analyzed by reverse phase chromatography, measuring the optical density at 214 nm. The positions of the elution peaks corresponding to insulin A- and B-chain as well as native insulin were indicated by subjecting their standard counterparts. The experiments were repeated 3 times. The standard A- and B-chain were also prepared from insulin of Sigma, following previous publication [30].

### Purification of natively folded human insulin

Ammonium sulfate was added to the supernatant containing the active insulin to a final concentration of 500 mM. Then, the solution was passed through a 0.22 μm filter (Biofil), and subjected to phenyl sepharose (GE Healthcare) column (1 × 20 cm, Pharmacia) at about 20 mg per mL resin. The column was connected to a Pharmacia liquid chromatography system, pre-equilibrated with 20 mM Tris-HCl, 500 mM ammonium sulfate at pH 8.0. The equilibration buffer was also used to wash the bound materials. Then, insulin was eluted with a descending linear gradient of ammonium sulfate (500–0 mM) in 20 mM Tris-HCl, pH 8.0. The experiment was done at flowrate of 1 mL/min with a fraction size of 2 mL and the eluates were analyzed by measuring the optical density at 276 nm. In the elution profile, the position of native insulin was identified by the addition of an authentic sample (standard human insulin). The elution with the same condition was considered for the natively folded insulin. The fractions corresponding to the native insulin were collected and dialyzed extensively against acetic acid (1 M) at 4°C [31, 32].

### Comparing the natively folded human insulin with a standard insulin

#### RP-HPLC analysis

For comparing the surface features of the recombinant and standard insulins, these two proteins were separately subjected to RP-HPLC column at 25°C (ProntoSIL 200-5-C18, 250 × 4.6 mm; Apex Scientific) equipped with UV detector (Smartline 2500; KNAUER). The insulin samples were chromatographed at a flow rate of 1 mL/min with a linear gradient of acetonitrile (24–60%) for 10 min at a constant temperature 25°C.

#### The fluorescence assessments

The intrinsic fluorescence was recorded in 50 mM sodium phosphate buffer pH 7.4, using an Agilent fluorescence spectrophotometer (Varian Cary Eclipse, USA). In order to measure the fluorescence of tyrosine (Tyr) residues, the insulin samples (1 mg/mL) were excited at 276 nm and the emission spectra were collected at the wavelength range of 280–400 nm. The excitation/emission band passes for Tyr fluorescence were set at 5/5 nm[33]. Also, bis-ANS fluorescence for the surface hydrophobicity assessment of the protein samples was performed [34]. The scans were average of three replicates.

#### Circular dichroism measurement

The CD measurements were performed in 50 mM sodium phosphate buffer pH 7.4 on a Jasco J-720 spectropolarimeter. The spectra were recorded in a 0.1 cm path length cell from 250 to 190 nm. The background CD spectrum of the buffer was subtracted from the CD spectra of the insulin chains. The reported spectra are the average of three scans [35]. The protein concentration was adjusted to 2 mg/mL by UV absorption at 276 nm using an extinction coefficient of 1.08 for 1.0 mg/mL. The CD experiments were done at room temperature.

#### Near infrared (NIR) spectroscopy assessment

Recombinant and standard insulin were analyzed by near infrared (NIR) Spectroscopy. The NIR spectra were collected on a NIRS XDS series Vis-NIR spectrometer (Metrohm, Switzerland) with the reflectance mode, in the range of 8,000 to 4,000 cm^-1^ and a resolution of 8 cm^-1^ at room temperature. For each dried sample, the NIR spectra were recorded through the bottom of the glass plate. To decrease the possible effect of uneven distribution of dried insulin, the protein samples were rotated and measured again [36]. Each spectrum consisted of the average of 3 replicates.

#### Insulin aggregation analysis

The aggregation of insulin (0.5 mg/mL) was induced in the presence of 20 mM dithiothreitol (DTT) in phosphate buffer (50 mM, pH 7.2) [37]. The kinetic of aggregation was assessed at 40°C for 20 min by recording the optical density at 360 nm, using a T90^+^ UV-Vis spectrophotometer (PG Instruments) which equipped with Peltier temperature controller. Also, formation of amyloid fibril was monitored by ThT fluorescence analysis [38]. Fibril formation was initiated by incubation of insulin (2 mg/mL) in 20% acetic acid pH 2.0, containing 150 mM NaCl for 5 h, at 60°C [39, 40]. At the end of incubation, 10 μM ThT was added to the protein solution and fluorescence measurement was done using an excitation wavelength at 450 nm [30]. The excitation and emission slits were 5 and 10, respectively.

#### The size exclusion chromatography assessment

The standard and recombinant insulin samples (2 mg/mL) were individually dissolved in Tris HCl (10 mM, pH 7.4), containing 0.11 mM ZnCl_2_ [41]. Under this condition, the molar ratio of insulin to zinc was about 3, favoring formation of the protein hexameric form [42, 43]. In this study, a KNAUER HPLC system equipped with the analytical size exclusion column (300 × 8 mm SEC column PSS SUPREMA) and DAD 2.1 UV detector was used. During the experiments, the temperature of HPLC column was also kept at 37°C. The HPLC results were average of three independent repetitions.

#### The insulin tolerance test (ITT)

For activity assessment of the natively folded fraction of final product, the insulin tolerance test (ITT) was performed in fasted mice [27]. Inbred *BALB/c* male mice between twelve to fifteen week old mice (weighing 22–27 g) were randomly divided into three groups of 7 mice each. The food was taken away 6 h before the start of the test. The control group received a subcutaneous saline injection (0.1 mL per 100 g body weight) while the experimental groups received a saline injection containing the standard and recombinant insulin. The samples were injected subcutaneously at a dose of 0.75 U/kg based on the unit of standard insulin [27]. One unit (U) of insulin is defined as 36.3 μg of the polypeptide. Blood glucose was measured with a glucometer (Accu-Chek Performa, Germany). To determine a basal glucose level, blood glucose was measured in a drop of blood obtained from the tail vein before the application of the samples. After injection of insulin product or saline, blood glucose measurements were done at time intervals of 10, 20, 30, 40, 60, 90, 120, 150 and 180 min. Before injection, blood glucose measurements were done for 60 min. The mice were given free access to food immediately after the experiment. All experiments followed the ethical guidelines for animal experiments were described and approved by the committee for experiments with laboratory animals of the National Research Ethics Committee. In this study, the mice were purchased from Shiraz University of Medical Sciences (Shiraz, Iran). The mice were fed normal chow which has been supplied in wire lid food hoppers, making the food and water easily accessible to them. Also the mice were housed at 23–24°C under a daily cycle of 12 h of light and dark.

### Statistical analysis

The data were analyzed in GraphPad Prism and presented as mean ±S.E.M. The significance of data was calculated using a two-tailed t-test for independent samples [27].

## Results

### Designing an appropriate fusion partner for individual expression of insulin A- and B-chain in *Escherichia coli*

In order to increase the expression level in *Escherichia coli*, we fused the A- and B-chain of human insulin to the αB-Cry gene. The internal methionine (residue 68) in wild-type αB-Cry was mutated to isoleucine in order to prevent the formation of additional unwanted cleavage products in the presence of CNBr that would otherwise need to be removed from the final product (**Fig 1**). The Asp-Pro sequence (residues 129 and 130) in αB-Cry is sensitive to the cleavage by formic acid and this reagent is needed for releasing of the insulin chains from the fusion proteins in the cleavage step. Therefore, Pro 130 was mutated to valine (**Fig 1**).

Also, design of the restriction site of *Nde*I (5'CATATG3') incorporated a methionine residue at the carboxy terminal end of human αB-Cry, at the boundary between the carrier protein and the desired peptide. Therefore, the expressed fusion protein can be cut at the boundary by cyanogen bromide treatment. Finally, the engineered expression plasmids were constructed and their sequence precision was confirmed (**Fig 1**). Hence, the final constructs contained the N-terminal αB-Cry partner, a CNBr cleavage site and the C-terminal A- or B-chain of human insulin. In addition, the construct for A-chain (αB-AC) contained an N-terminal hexa-histidine tag to enable rapid and straightforward purification. The details of these constructs are given in **Fig 1**.

### Expression and purification of the fusion proteins

**Fig 2:**
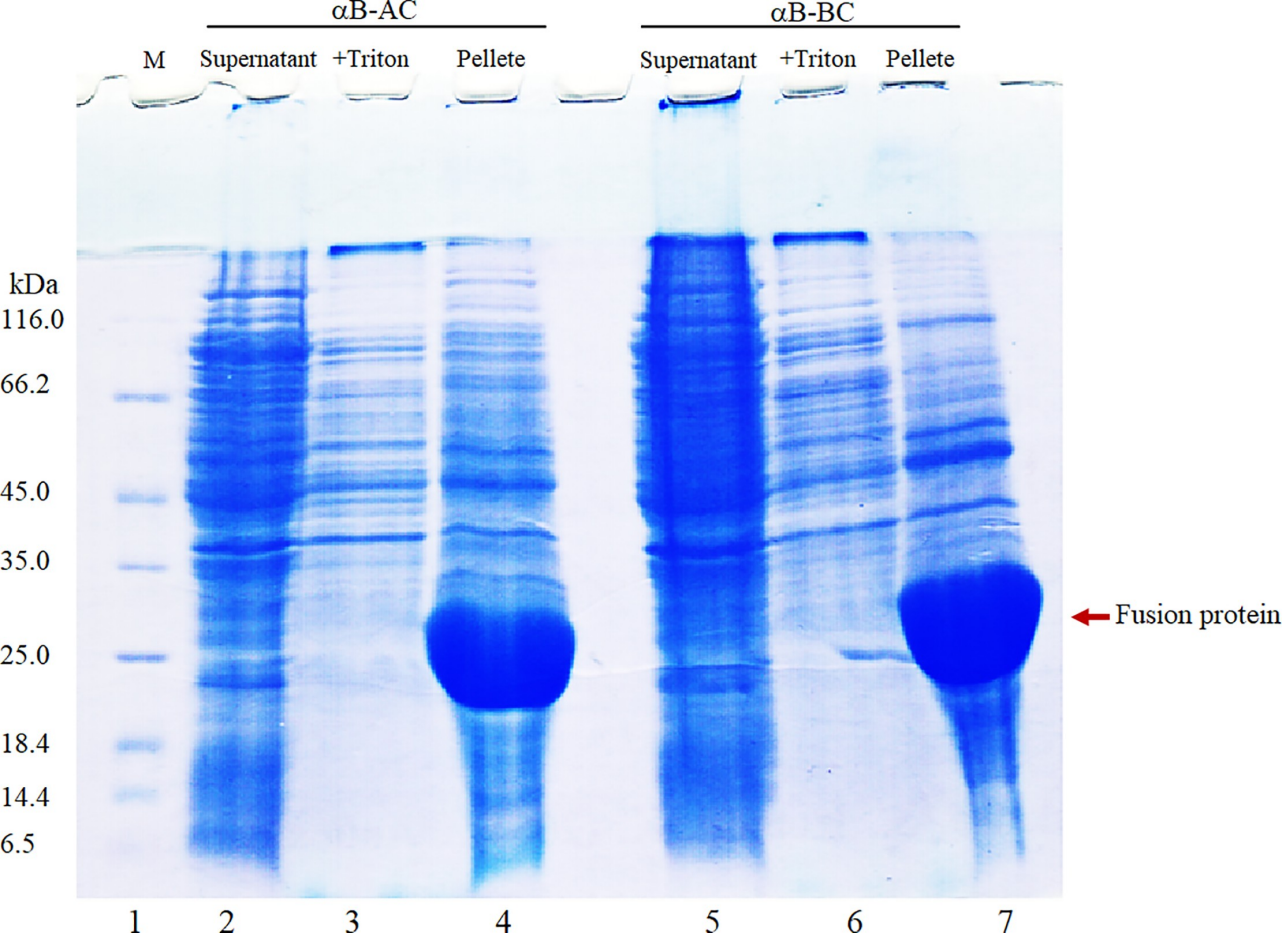
The SDS–PAGE analysis of the fusion proteins expression and purification. The protein samples in different steps of the purification of fusion proteins (αB-AC and αB-BC) were subjected to gel electrophoresis analysis. Lane 1 indicates molecular mass marker. Lanes 2, 3 and 4, respectively, stand for the supernatant, the pellet subjected to the subsequent washing with Triton X-100 and urea, and αB-AC fusion protein solubilized in 8 M urea. Lanes 5, 6 and 7 indicate SDS-PAGE analyses of protein in the similar steps during purification of αB-BC fusion protein.

The desired constructs were transformed to XL1blue cells. Following extraction and sequencing, the vectors were then transformed to BL21 cells. BL21 cells harboring recombinant plasmids pET28b (+) were induced separately to express the αB-AC and αB-BC fusion proteins, using IPTG inducer. The SDS–PAGE analysis suggested that the expressed fusion proteins were completely insoluble and supernatant of the cell lysates after sonication had no obvious desired proteins (**Fig 2**, lanes 2 and 5). Based on SDS-PAGE results the expressed fusion proteins (αB-AC and αB-BC) were constituted approximately 31% of the whole cell extract with the complete partitioning into the insoluble fraction (**Fig 2**). The inclusion bodies containing the αB-AC and αB-BC were isolated by a low speed centrifugation. As a pre-step purification, the partially soluble contaminating proteins were removed by Buffer A (**Fig 2**, lanes 3 and 6). Also, a solution of 8 M urea was used for solubilization of the fusion proteins (**Fig 2**, lanes 4 and 7). The solubilized inclusion bodies contain approximately 48.1% and 54.4% of the αB-AC and αB-BC, respectively.

**Fig 3:**
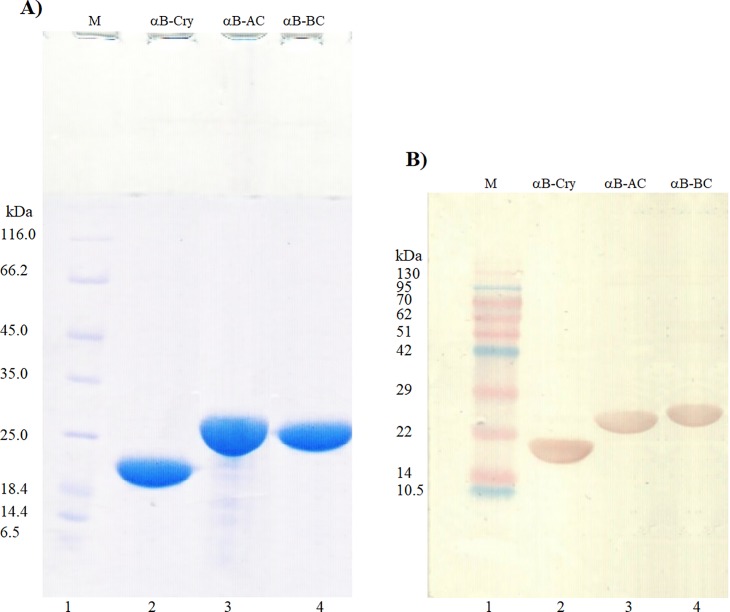
SDS–PAGE and western blot analyses of αB-Cry and the fusion proteins. The fusion proteins were analyzed by reducing SDS-PAGE (gel 12%) (**A**), and western blot (**B**). Lane 1 shows molecular mass marker. Also, lanes 2, 3 and 4, respectively, indicate αB-Cry, αB-AC and αB-BC. In the western blot analyses, the antibody against αB-Cry was used.

The solubilized inclusion bodies were further purified by a one-step anion exchange chromatography, using DEAE matrix (for αB-BC) and immobilized metal affinity chromatography by Ni-NTA column (for αB-AC) (**Fig 3A**, lanes 3 and 4). Eventually, using these methods, about 130 and 170 mg of the αB-AC and αB-BC were obtained from 1 L of the LB medium. The purified fusion proteins have an apparent molecular weight of 23–25 kDa (**Fig 3A**) which is corresponding to the sum of molecular mass of the αB-Cry fusion partner (20 kDa) and insulin A-chain (2.4 kDa) or B-chain (3.4 kDa).

As shown in the **Fig 3A**, the expressed fusion proteins appeared highly pure. In addition, to confirm the identity of the purified bands, the fusion proteins were detected by western blot analysis which has been developed with an anti αB-Cry antibody recognizing partner protein in the fusion molecules (**Fig 3B**, lanes 3 and 4).

### Cleavage of the fusion protein, sulfitolysis and isolation of S-sulfonated insulin chains

**Fig 4:**
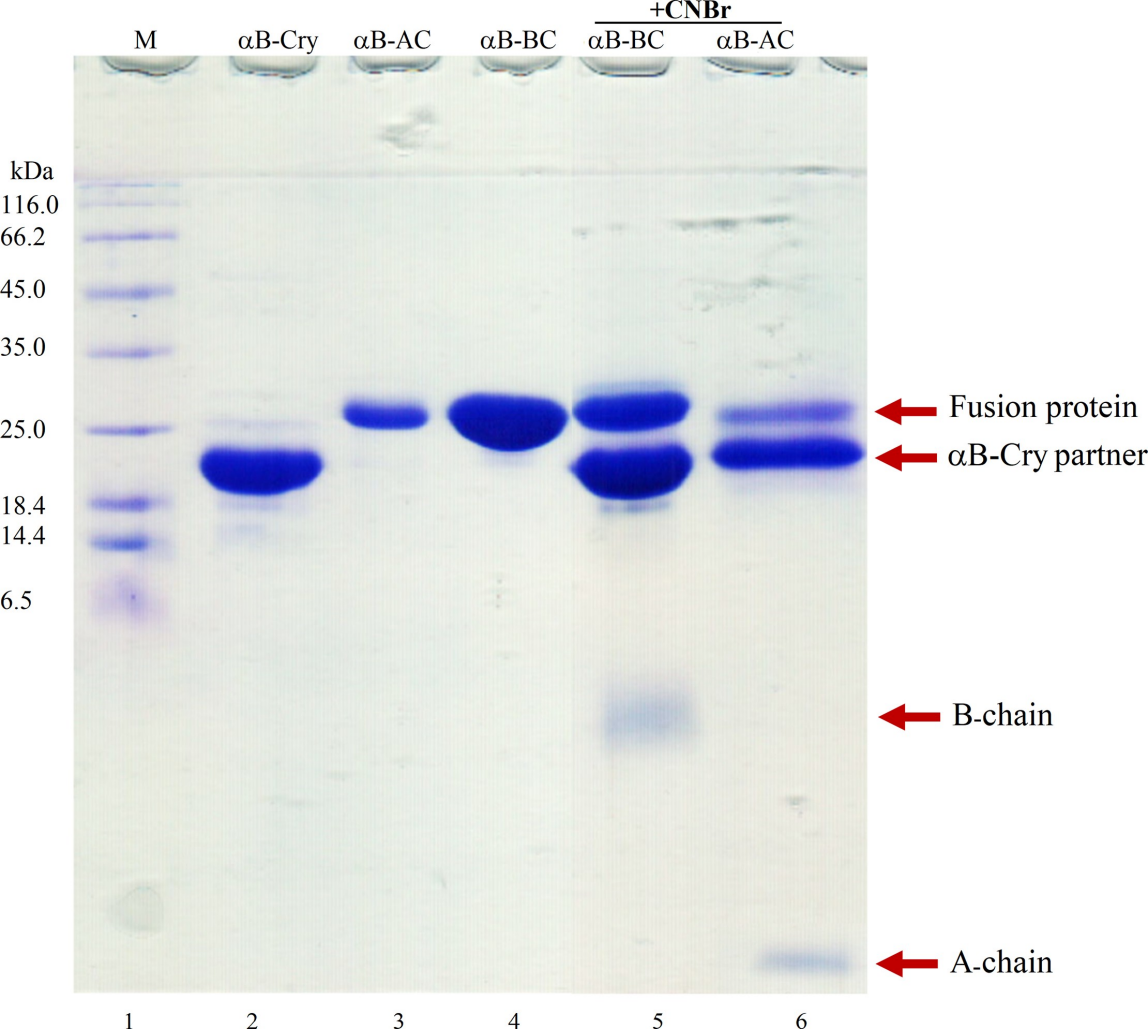
SDS–PAGE analyses of the fusion proteins cleavage. The fusion proteins were subjected to CNBr cleavage and the protein samples were analyzed by reducing SDS-PAGE (gel 18%). Lane 1 stands for the molecular mass marker. Lanes 2, 3 and 4 indicate αB-Cry, αB-AC and αB-BC, respectively. Lanes 5 and 6 show αB-BC and αB-AC fusion proteins after cleavage by the CNBr.

After purification of the fusion proteins, the αB-Cry carrier was efficiently removed by the action of a site-specific cleaving reagent. As shown in **Fig 4** after 24 h incubation with CNBr, the fusion proteins were cleaved to the significant level. At this stage three fragments including αB-Cry partner protein (20 kDa), insulin A-chain (2.4 kDa) or B-chain (3.4 kDa) and trivial quantity of uncleaved fusion protein (~25 kDa) were detectable on the SDS-PAGE gel (**Fig 4**). Quantitative comparison of the digest efficiency, using Image J software, revealed the significant CNBr cleavage of the fusion protein. The cleavage of αB-BC and αB-AC was estimated to be approximately 68.8 and 75.2%, respectively **(Fig 4**, lanes 5 and 6). The efficient cleavage occurred within 24 h with a CNBr to methionine molar ratio of 100:1 at room temperature. Increasing the incubation time or amount of CNBr did not improve the cleavage efficiency. Chemical cleavage experiment demonstrated that the full length fusion protein bands appreciably disappeared giving rise to the new bands of the smaller sizes corresponding to fragments αB-Cry (20 kDa) and insulin A- or B-chain. Prior to separation of the insulin peptides from their corresponding fusion proteins oxidative sulfitolysis which prevents improper disulfide binding, was performed. This experiment was done by adding sodium sulfite and sodium tetrathionate to the solutions containing insulin chains as described in the experimental section.

**Fig 5:**
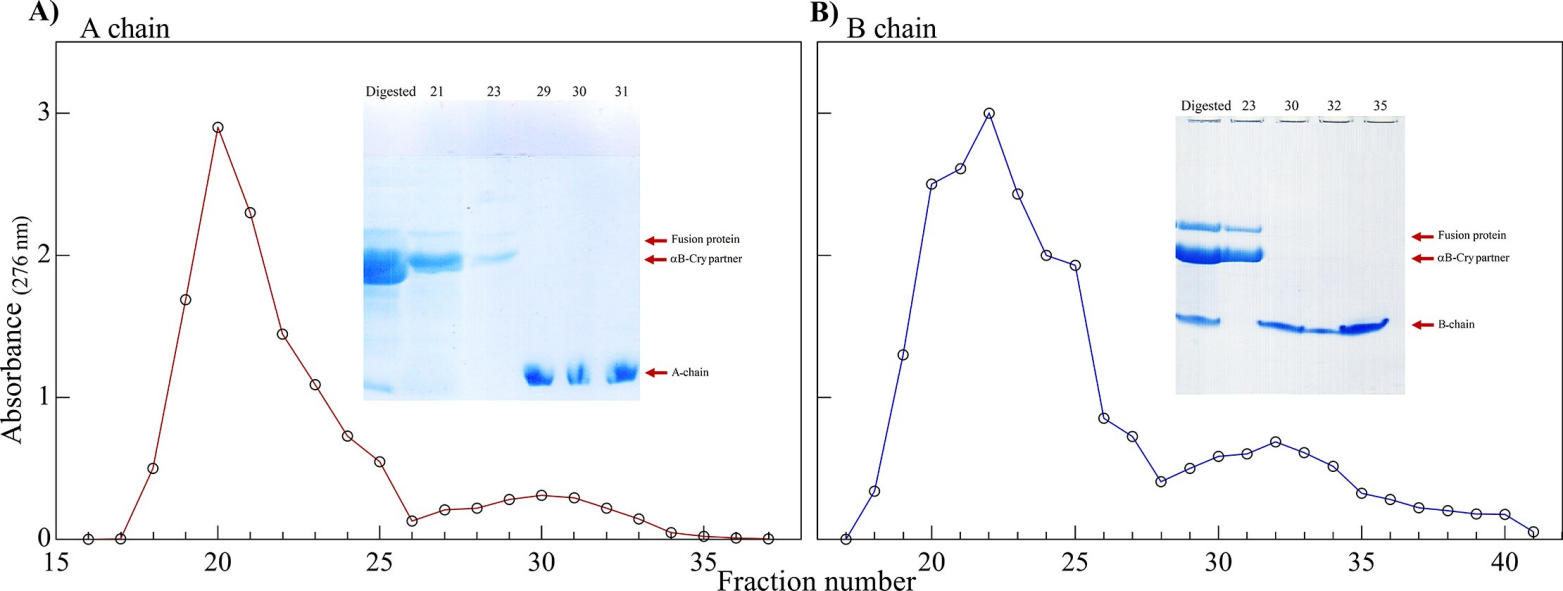
Gel filtration and SDS-PAGE analyses of the digested fusion proteins. The digest products of two fusion proteins with CNBr were individually applied to a Sephadex G50 gel filtration column (1.6 x 94 cm). The experiments were done by dissolving αB-BC in 1 M acetic acid and αB-AC in 20 mM Tris containing 8 M urea at pH 8.0. The elution profile and reducing SDS-PAGE profile (gel 18%) (the insets) corresponding to αB-AC (**A**) and αB-BC (**B**) are indicated.

After that, using a single-step gel filtration chromatography, the digested mixtures were separated into two peaks (**Fig 5**). The eluted fractions were then run on SDS–PAGE (insets of **Fig 5**). In **Fig 5A** and **5B**, the fractions eluted as the first peak contain undigested fusion protein and αB-Cry. Also, the fractions eluted as the second elution peak which belong to the insulin chains were collected and concentrated. After confirming suitable purification of the insulin peptides from residual fragments, the peptide chains were dialyzed twice against double distilled water (pH 5.5) and then lyophilized.

**Fig 6:**
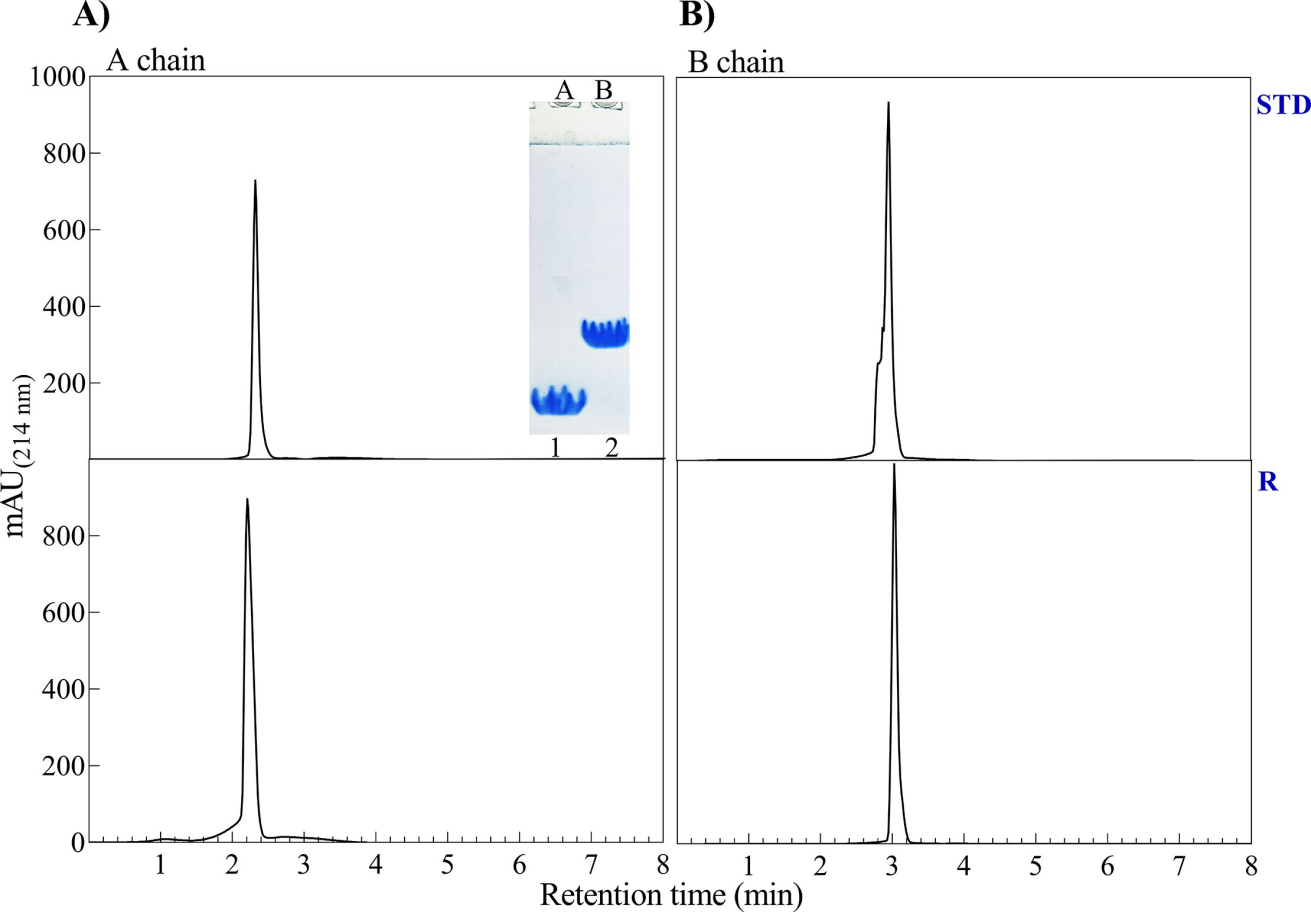
**RP-HPLC and SDS-PAGE analyses of A- and B-chain of human insulin.** The purified samples of A- and B-chain of human insulin were subjected to RP-HPLC and SDS-PAGE analyses. RP-HPLC analysis was conducted on C18 analytical column for the A- and B-chain of both standard insulin (STD) and human recombinant insulin product (R). The inset also shows the SDS–PAGE profile (gel 18%) of the purified chains of human insulin.

Finally, to verify the efficiency of our purification methods and to validate the identity of the recombinant A- and B-chain, purified and lyophilized peptides were analyzed by SDS–PAGE (gel 18%) and analytical RP-HPLC (**Fig 6**). Then, the dialysis against pure water at pH 5.5 results in precipitation of insulin A- and B-chain. Under such condition, the insulin chains are known to be highly insoluble whereas other possible contaminations were appeared in the soluble phase. The migration pattern of insulin A- and B-chain in the SDS–PAGE gel is also indicated in the inset of **Fig 6**.

Since the insulin peptides have small molecular masses than the fusion partner, the highly pure peptides could be obtained by gel filtration chromatography without needing a help of other kinds of affinity tags or high-resolution chromatographic methods, such as HPLC. Approximately, 27.6 mg and 43.2 mg of the purified S-sulfonated A- and B chain were generally obtained from 500 mg of the digested mixture.

Analytical RP-HPLC indicated the presence of a single major component at appreciate purity (**Fig 6**). Also, using standard A- and B-chain and measuring their retention time of elution, the identity of the recombinant peptides was analyzed. Our insulin peptides were highly pure and eluted synchronously with the corresponding peptide chains of the standard insulin (**Fig 6**).

### Applying human αB-Cry to increase the efficacy of insulin chain combination and folding

**Fig 7:**
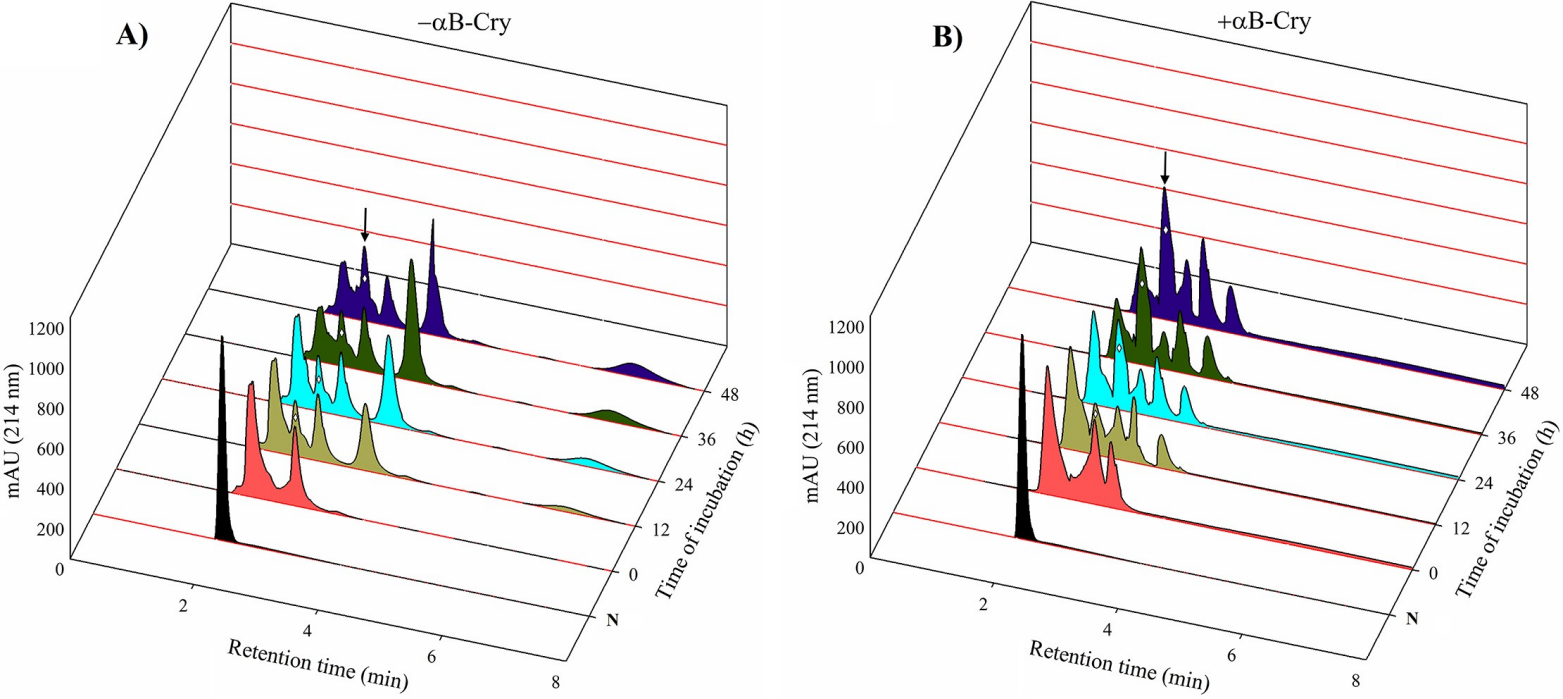
The chain combination analysis with RP-HPLC in the presence of αB-Cry. αB-Cry chaperone was used to increase efficacy of the chain combination step. (**A**) Peaks 1–4, respectively, indicate the position of oxidized A-chain, native insulin, oxidized B-chain and intermediate products presumably with the incorrect disulfide linkage. (**B**) Peaks 1–5 stand for the oxidized A-chain, native insulin, oxidized B-chain, αB-Cry and presumably the intermediate insulin species with the incorrect disulfide linkage. In both (**A**) and (**B**), the peaks correspond to the native insulin are distinguished by the white solid diamond as indicated by authentic insulin in the (**N**) line. The experiments were repeated three times.

As shown in **Fig 7**, the chain combination experiment was conducted by the incubation of A- and B-chain of human insulin with a weight ratio of 2:1, respectively, for 48 h. As revealed by the analytical RP-HPLC analysis, in the absence of appropriate chaperone, approximately after 12 h of incubation, the formation of natively folded human insulin molecules was reached to its steady state (**Fig 7**). As shown in **Fig 7**, the usable chains available for the further combination and pairing were also aggregated at the initial steps of the folding process, giving rise the amount of insulin aggregates species. Spending more times had no significant effect on the formation yield of insulin molecules with native folding.

As shown in **Fig 7A**, at 12 h interval, some undesirable intermediates were developed. Due to their longer retention time, the incorrect species may present at the high hydrophobic state. Formation of these intermediates is possibly due to the wrong disulfide pairing or hydrophobic collapse of insulin A- and/or B-chain. At this condition, the amount of A- and B-chain of human insulin can be gradually decreased and this reduction was accompanied with the increment in the quantity of insulin misfolded intermediates. To solve this problem, we applied human αB-Cry to increase the yield of insulin chain combination and folding (**Fig 7B**). As shown by the analytical RP-HPLC analysis the joining of the S-sulfonated A- and B-chain to form natively folded and active hormone, as assisted by the chaperone (αB-Cry), gives a significantly improved yield. Moreover, the incorrect protein species, indicating longer retention time, were fully disappeared as αB-Cry was used to assist the chain combination and folding of the human recombinant insulin.

Based on the results of respective RP-HPLC profiles, during 48 h of the incubation, the A- and B-chain in the presence of αB-Cry remain largely soluble to have further chance of the correct pairing which subsequently increase the quantity of natively folded insulin molecules. However, in the absence of αB-Cry, the amount of insulin chains was gradually reduced without generating native insulin molecules (peaks that marked by diamond correspond to native insulin). The efficiency of insulin chain combination and folding during the mentioned times were calculated from the total and corresponding peak areas. The efficiency in each incubation time is indicated in **Table 1**. In particular, by developing the incubation time in the presence of αB-Cry, the area corresponding to the insulin molecules with correct folding was remarkably increased (**Fig 7B**, diamond marked peaks). It should be noted that with the increase in the intensity of the peak for native insulin, the intensity of the peaks corresponding to the A- and B-chain was decreased. However, the samples which were exposed to folding conditions in the absence of αB-Cry, did not show significant differences in the peak corresponding to native insulin with the respect to the increase in incubation time. It should be noted that the yield was calculated on the basis of the amounts of all products for each incubation time.

**Table 1:** The refolding efficiency (%) of recombinant human insulin.

Time of incubation (h)	- αB-Cry	+ αB-Cry
12	5.0 ± 1.32	6.3 ± 0.86
24	7.8 ± 1.03	11.7 ± 1.05
36	8.0 ± 0.91	19.3 ± 1.79
48	10.2 ± 1.11	26.7 ± 2.02

**Fig 8:**
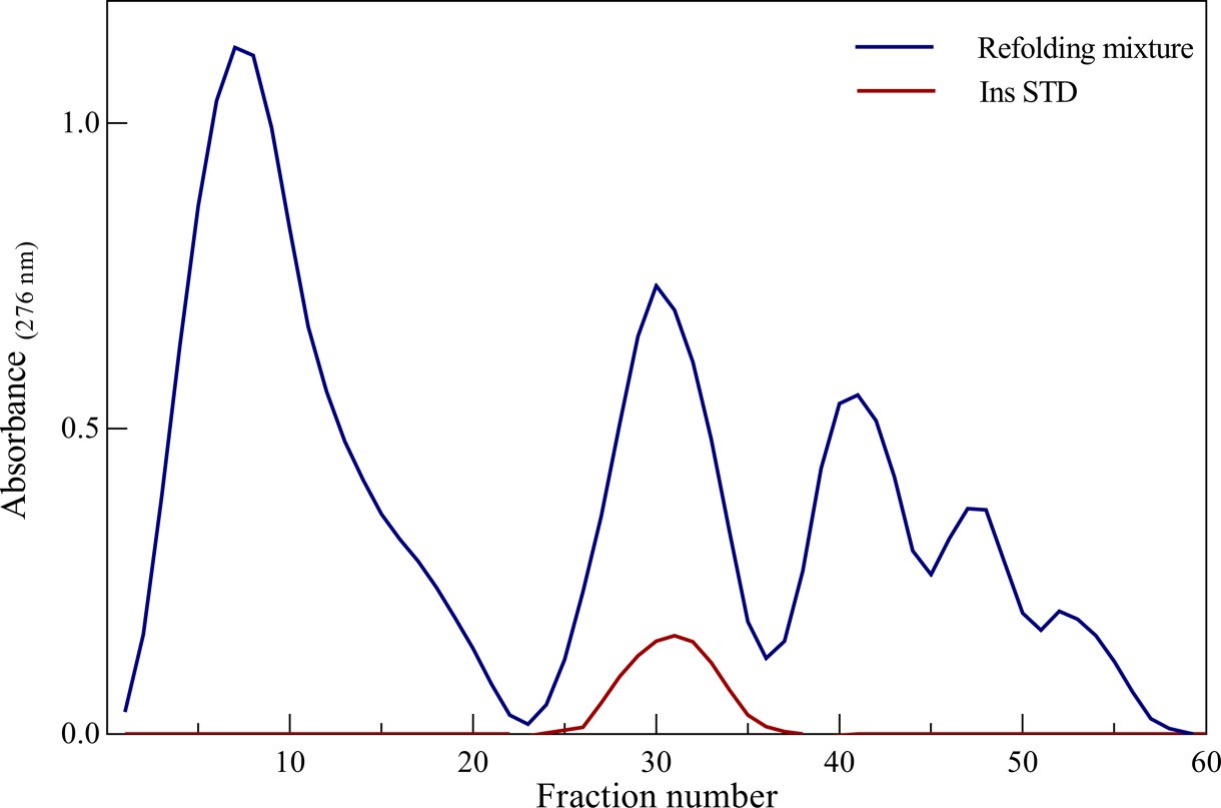
Purification of the insulin peptide chains on phenyl sepharose column. The natively folded human insulin was purified using hydrophobic interaction chromatography (HIC) on a phenyl sepharose column. The elution was achieved with a reverse linear gradient of ammonium sulfate (500–0 mM) in 20 mM Tris HCl, pH 8.0. The standard insulin in the similar condition was also subjected to the same column for comparing the elution profile of the natively folded human recombinant insulin.

Hydrophobic interaction chromatography (HIC) is commonly used to separate natively folded and unfolded species during the purification of therapeutic proteins. In general, the separation of proteins by HIC largely depends on the hydrophobicity of protein, the temperature and the properties of the stationary and mobile phases. In this system, the elution times are related to hydrophobic contact area, therefore an increase in what affecting surface hydrophobic area of the sample results in increased binding to HIC media [44]. For purification of natively folded recombinant insulin, HIC with a phenyl sepharose hydrophobic column was used. To perform this experiment, a mixture of BSSO3^-^ (40 mg) and ASSO3^-^ (80 mg) which has been incubated with αB-Cry (αB-Cry to B-chain molar ratio of 0.04) for 48 h was used (**Fig 8**). By reducing the concentration of ammonium sulfate, a mixture of misfolded intermediates was eluted at the initial step of the purification process (**Fig 8**). In this purification step, 27.08 mg of the human recombinant insulin, similar to the standard insulin, was obtained which corresponds to a yield of 22.57%. As authentic insulin was subjected to the same column, it displayed a similar elution time to that of natively folded human recombinant insulin. It should be noted that, as far as the elution time is concerned, our recombinant human insulin product and standard insulin of Sigma was eluted closely at the similar concentration of ammonium sulfate (250 mM).

### Structural and functional characterization of the human recombinant insulin product

#### Analytical RP-HPLC for validation of native folding of the recombinant insulin

**Fig 9:**
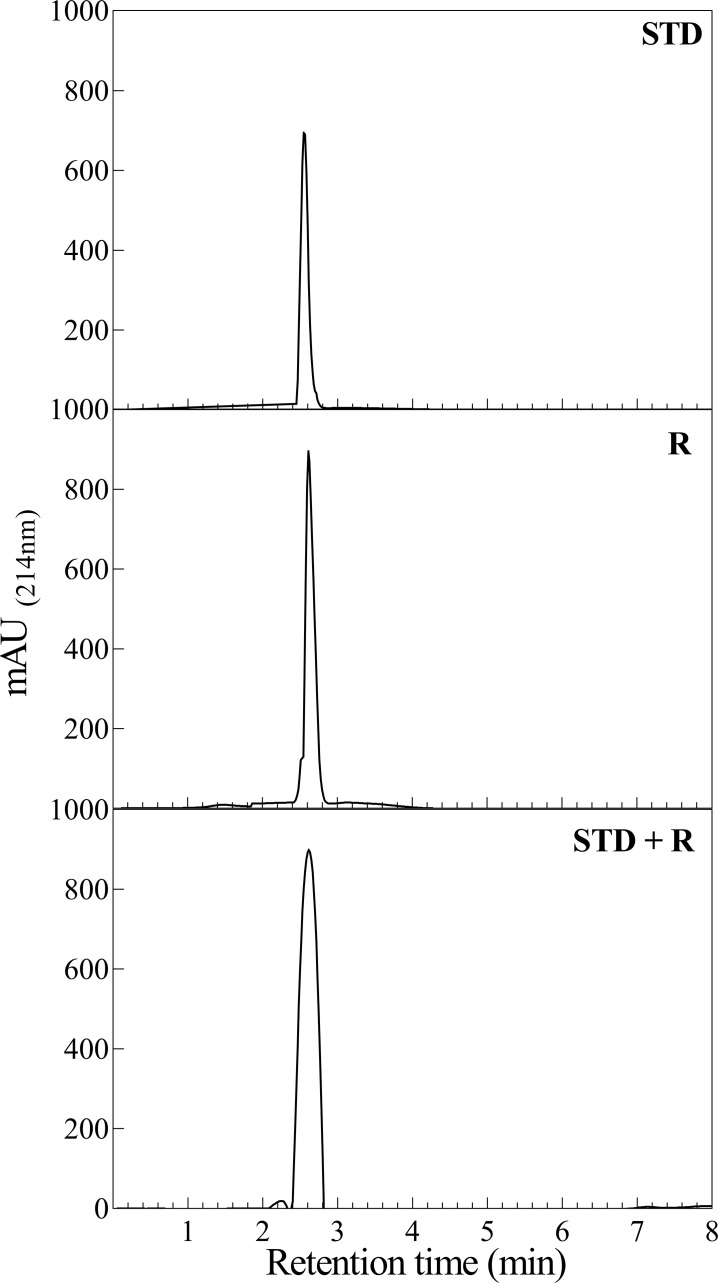
Comparing the RP-HPLC elution profile of the two insulin. The human recombinant insulin was compared with standard insulin based on their elution profiles on the analytical RP-HPLC using C18 column.

The lyophilized powder of purified insulin product obtained from the phenyl sepharose column was dissolved in 0.5 mL acetic acid (20%), filtered through 0.22 μm filter and subjected to a hydrophobic C18 analytic column. As shown in **Fig 9**, to confirm the surface identity of our recombinant insulin product, the standard insulin was also individually subjected to the HPLC column, under similar conditions. As indicated in **Fig 9**, human insulin product and standard insulin displayed similar retention time on this column.

It is essential to note that the standard insulin and our insulin product might be in their monomeric state as they dissolved in a solvent containing 20% acetic acid [40]. When recombinant and standard insulin were mixed together and subjected to the same chromatography procedure, they were eluted as a single peak. Their similar elution profile on C18 column suggests their comparable structural folding and exposed surface characteristics.

#### Structural analyses of the human recombinant insulin product

**Fig 10:**
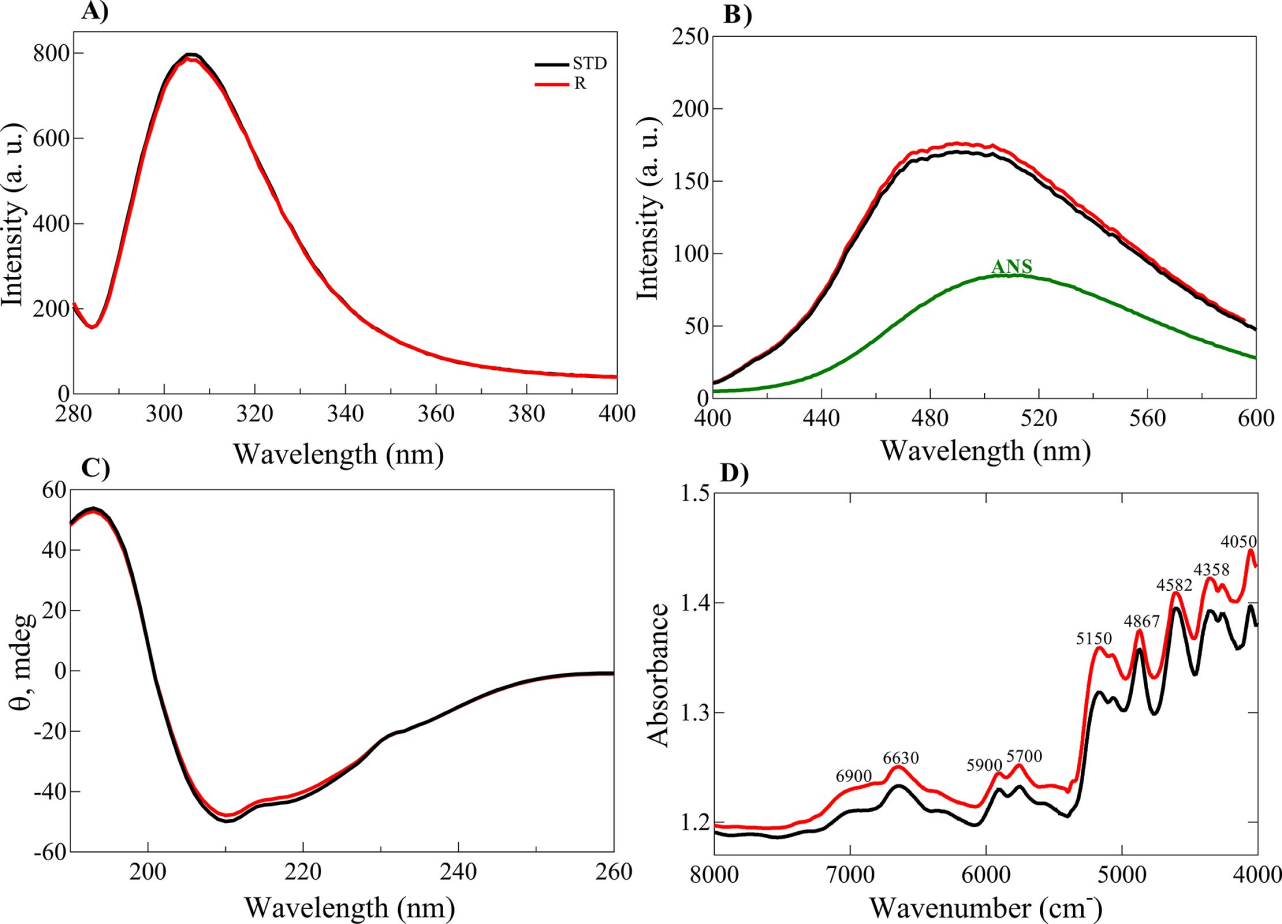
Structural characterization of natively folded human recombinant insulin. Tyrosine fluorescence emission spectra of the natively folded and standard insulin. The insulin samples (1.0 mg/mL) were excited at 276 nm, with emission scans from 280 to 400 nm. (**B**) The solvent exposed hydrophobic surface was detected by bis-ANS fluorescence. The protein samples (1.0 mg/mL) containing 10 μM bis-ANS were incubated at room temperature for 20 min. Upon excitation at 350 nm, the bis-ANS fluorescence spectra were measured from 400 to 600 nm. (**C**) The far UV-CD spectra of the insulin samples. The protein samples (2 mg/mL) were dissolved in phosphate-buffered (50 mM phosphate pH 7.4) and the CD spectra were scanned from 260 to 195 nm (**D**) The NIR spectra of powder of the two insulin are indicated. Band assignments are depicted in the figure.

The structure of our insulin product was compared with that of standard insulin using fluorescence, circular dichroism (CD) and near infrared (NIR) spectroscopic analyses. The highly sensitive technique of intrinsic protein fluorescence has been widely used to compare the structure of proteins. It has been previously indicated that the structural alteration of insulin can be directly detected by Tyr fluorescence measurement. Human insulin contains four Tyr residues, upon exposing and/or unfolding its molecular structure, the quantum yield of these residues is altered [33]. Tyr fluorescence analyses (**Fig 10A**) showed that the two insulin samples hold similar intensity. Also, the ability of bis-ANS to bind to the exposed hydrophobic patches of the two insulin samples indicated no significant difference (**Fig 10B**). The results of both Tyr and bis-ANS florescence study suggested that two insulin samples have appreciable structural similarity. Also, the secondary structure content of the two insulin samples was analyzed and compared by CD and NIR spectrometry. The far UV-CD spectra of both insulin samples (**Fig 10C**) are very similar, suggesting that they both adopted a similar α-helical secondary structure. Due to the high ratio of disulfide bonds existing in insulin, the CD signal at 251 nm has been previously used to monitor the conformation and overall disulfide bond pattern in this hormone [45, 46]. Judging from the CD signal at this wavelength, it is concluded that disulfide bond arrangement is similar in these two insulin samples.

The NIR spectra of these insulin samples were also collected (**Fig 10D**). Likewise, two other bands in the regions 5900–5700 cm^-1^ correspond to the first overtones of the C–H stretching. The band at 5,150 and 6,900 originate from the vibrations in the water molecules and the frequency depends on the environment and hydrogen bonding of the water molecules. These two peaks show that the insulin samples contain water molecule in their structures. The bands at the 5200–4000 cm^-1^ have been attributed to the combinations of amide I, amide II, amide III, amide A, amide B and C–H stretching bands [36, 47]. Overall, these bands correlated to the secondary structure of the insulin samples. In agreement with the results of CD analyses, the data of NIR also suggest that the secondary structures in two insulin samples are in the appreciable similarity. Overall, the results of spectroscopic analyses suggest that our insulin product revealed a substantial structural similarity with that of the standard insulin.

**Fig 11:**
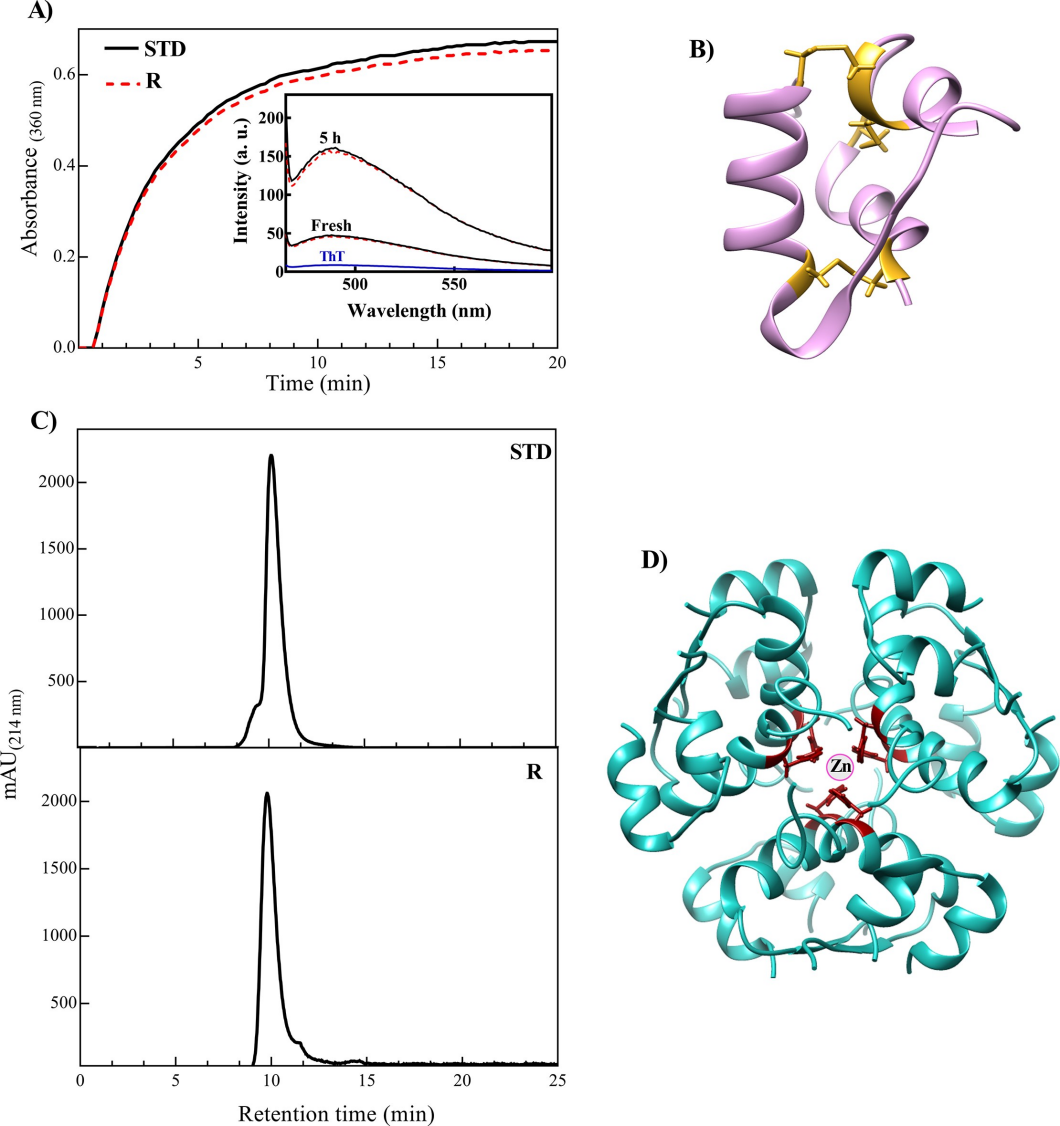
The aggregation and size exclusion analyses of the recombinant and standard insulin samples. **(A)** The tendencies of two insulin samples (0.5 mg/mL) were analyzed in the presence of DTT at 40°C. The **Inset** displays ThT fluorescence emissions of two insulin samples which incubated for 5 h under fibrillogenic conditions. **(B)** Schematic representation of insulin monomer extracted from 3AIY pdb. The disulfide bonds indicated in yellow. **(C)** The size exclusion chromatography was performed in a condition favoring formation of the hexameric form. The **STD** and **R** stand for standard and the recombinant insulin samples, respectively. **(D)** The hexameric form of human insulin extracted from 3AIY pdb and red residues show His^B10^.

The reduction of S–S bridges between A- and B-chain of insulin results in formation of the amorphous aggregates [37]. In the current study, the aggregation propensity of two insulin samples (standard and recombinant) were analyzed in the presence of DTT (**Fig 11A**). Our result suggests a similar aggregation pattern for these two insulin samples. The three dimensional structure of insulin monomer also displayed in **Fig 11B**. As reported previously, the disulfide bonds in insulin structure have specific accessibility to the environment which highly depend on the structural feature of this hormone [48]. Therefore, the similar aggregation pattern of two insulin samples is likely due to their comparable structures (**Fig 11A)**. The ThT has been previously used to monitor development of amyloid fibrils in proteins [30, 40]. After incubation of the insulin samples under fibrillogenic conditions, the ThT fluorescence measurement was done. The inset of **Fig 11A** shows a significant increment in the ThT fluorescence emissions, suggesting formation of the amyloid fibrils by the both insulin samples. Moreover, the two insulin samples indicated a very similar ThT emission profiles which may suggest a comparable folding in their native structures.

In this study, the oligomerization patterns of the two insulin samples were also compared by size exclusion chromatography (**Fig 11C**). As reported before, when the molar ratio of insulin to zinc is about 3, this peptide hormone at physiological pH and appropriate concentration is capable to appear in its hexameric state [42, 43, 49].

As shown in **Fig 11C**, and in agreement with previous study [50], the retention time of the standard and recombinant insulin samples were almost similar to each other. Therefore, these insulin samples are likely to be in their hexameric state (**Fig 11D**).

#### *In vivo* activity assessment of the insulin product

**Fig 12:**
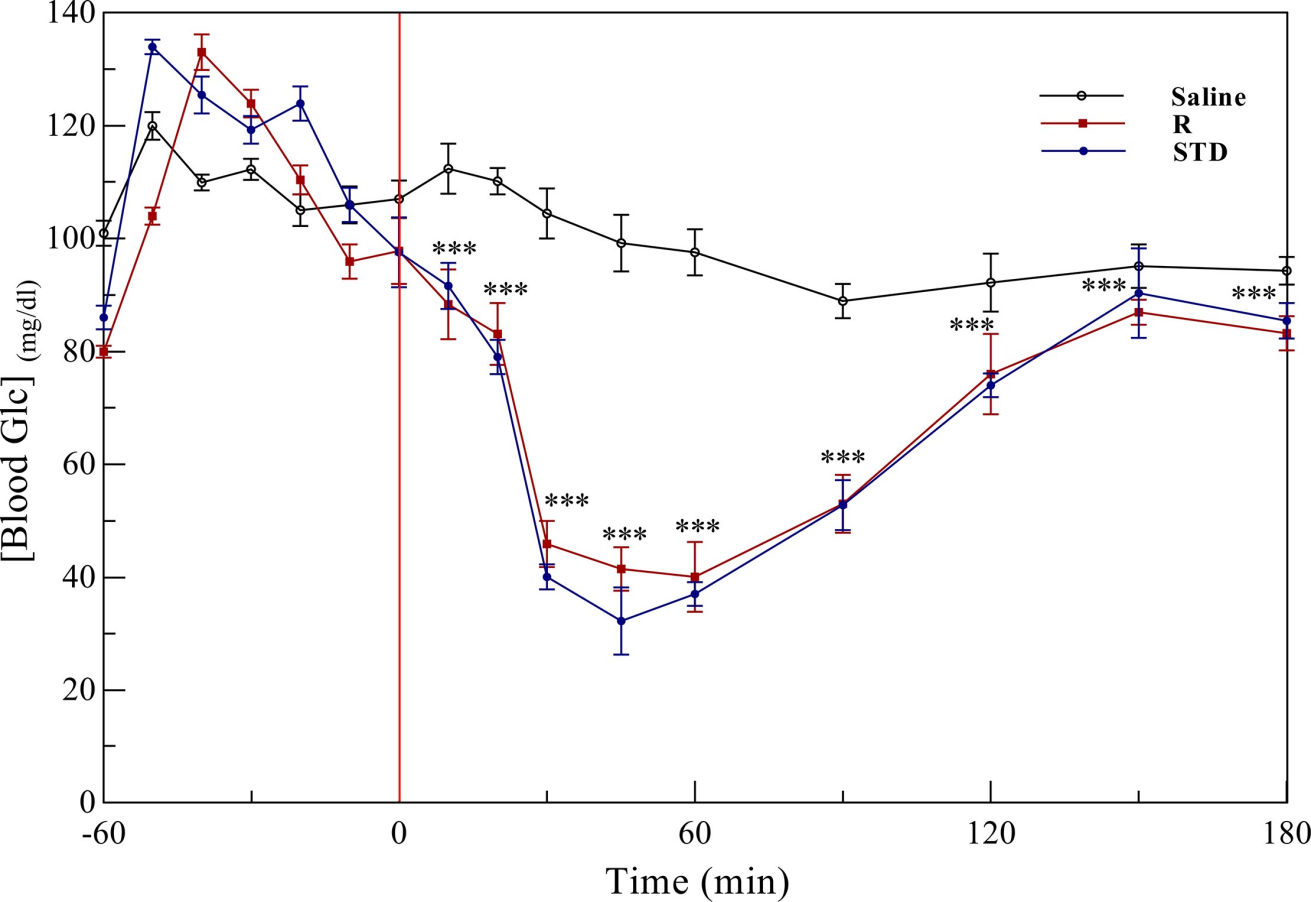
The insulin tolerance test in mice. Insulin tolerance test (ITT) was performed in mice with the age of 12 and 15 weeks, and average 25 g of their body weight. Values are mean ± S.E., n = 7/group. ***p<0.01 compared to human insulin treated group.

Though, for the *in vivo* activity assessment, insulin tolerance test (ITT) in mice was studied. An ITT is a procedure in which insulin is injected into the mice, then concentration of the blood glucose is measuring at the regular intervals [27, 51]. After subcutaneous injection of the insulin samples, a significant reduction in the blood glucose level was observed. Also, both standard insulin and our insulin product displayed a similar time-dependent effect on the level of blood glucose in mice (**Fig 12**). A negative control was also prepared by the injection of saline alone into mice.

Overall, our results suggest a similar pattern of glucose lowering activity for these two insulin samples.

## Discussion

**Fig 13:**
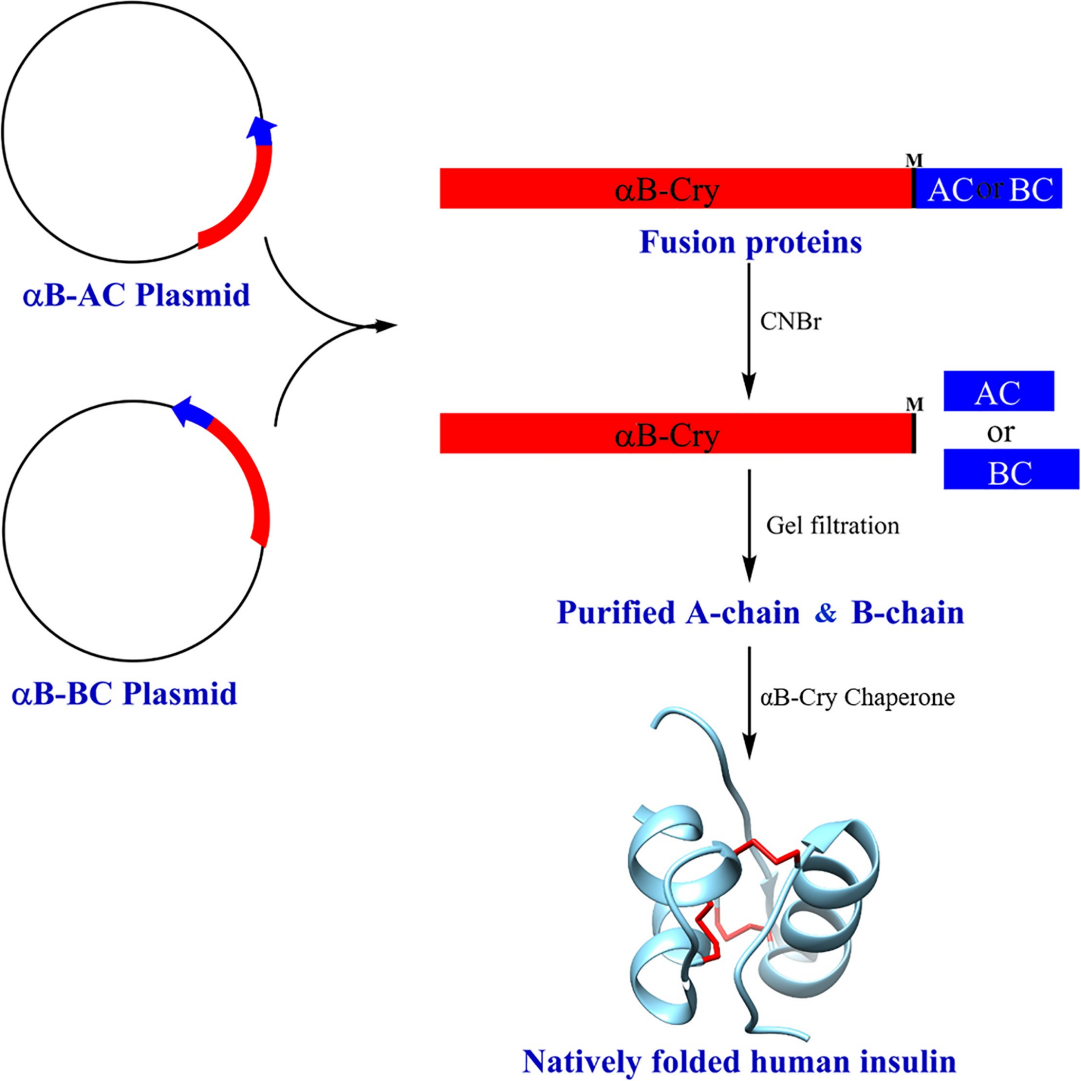
Schematic diagram of the experimental procedure. The constructs contain ATG codon for coding methionine (**M**) as the specific CNBr cleavage site. The fusion genes or proteins αB-Cry/A-chain and αB-Cry/B-chain indicated as αB-AC and αB-BC, respectively.

Although insulin does not require complex post-translational modifications its expression in *Escherichia coli* is challenging because its two peptide chains are highly susceptible to degradation by the host’s enzymes [52]. To overcome the difficulties associated with expressing insulin genes in *Escherichia coli*, a suitable fusion partner (tag) protein could ensure its stability in microorganisms and facilitate its purification. The role of a fusion partner is to sequester the fused peptide in the inclusion bodies, conferring resistance to its degradation, increasing the expression rate and simplifying the recovery of the small peptide [11, 53]. In the current study, when human αB-Cry was selected and designed as a fusion partner for high expression level of the insulin chains, the following beneficial factors were considered. First, the size of αB-Cry (175 amino acids) is ~6 times smaller than the β-gal fusion protein partner (1000 amino acids) [16], which subsequently increases the insulin production yield to a significant level. Second, αB-Cry has an intrinsic ability to form large oligomers in solution [21, 54, 55], and therefore, it may provide an easier condition to separate the A- and B-chain from their fusion partners by a suitable size exclusion protocol. Third, the αB-Cry fusion partner lacks cysteine, therefore, it will not engage in disulfide bonding with the six cysteine residues in two peptide chains of human insulin. Fourth, the bacterial expression host may take the advantage of the chaperone activity of the carrier protein (αB-Cry) which may provide an intrinsic tolerance to overcome harmful environmental stresses [56]. Moreover, according to the results of previous studies, α-Cry promotes the dissociation of insulin oligomers to the lower associated species (dimers and monomers) [42] and displays a superior stabilizing effect on insulin. A chemical cleavage site (CNBr cleavage site) at the end of α-Cry carrier protein provides a convenient condition for recovering the target peptides from the digested fusion proteins (αB-AC and αB-BC). We also took advantage of the chaperone activity of this carrier protein to increase the yield of *in vitro* chain combination and folding of human recombinant insulin. A summary of our expression, purification and folding strategy is shown as **Fig 13**.

Based upon the above information, αB-Cry fully meets the requirements as a suitable carrier protein and chaperone molecule for expression of the insulin chains. Moreover, this chaperone has been already suggested as a suitable excipient candidate in the formulation of therapeutic insulin [42].

The SDS-PAGE results suggested that the fusion proteins (αB-AC and αB-BC) were fully expressed as inclusion bodies in *Escherichia coli* (**Fig 2**). The intrinsic chaperone ability of αB-Cry through the intermolecular association of hydrophobic domains may facilitate the sequestering of the fusion proteins in the inclusion bodies (**Fig 2**) [57, 58]. Cell lysis followed by washing of the inclusion bodies led to the fusion proteins with a purity more than 48%. The fusion proteins were then purified using a single-step purification approach which produced these proteins in high yield and reasonable purity. The CNBr release of insulin peptide chains from the fusion proteins was also efficient (68.8% and 75.2% for αB-BC and αB-AC, respectively). The fusion proteins indicated high solubility in the CNBr digesting solution and this chemical cleaving agent was also simply removed from the digested mixture by dialysis. Using a single-step gel filtration, we purified the A-and B-chain of human insulin (**Figs 5** and **6**).

Subsequently, combination of these chains to form active insulin was our main goal. Six cysteine residues are present in the A- and B-chain, giving rise to three disulfide bonds [59]. Therefore, statistically, there are 15 possibilities of forming disulfide bridges within one human insulin molecule and only one giving rise to native functional human insulin with the following disulfide bridges: A6-A11; A7-B7 and A20-B19 [60]. Fortunately, previous studies achieved correct folding of insulin molecules in the presence of a mixture of S-sulfonated A- and B-chain under a reducing environment [31]. However, Dixon and Wardlaw indicated an approximate yield of only 2% for the chain combination of the oxidized insulin peptide chains [20, 61]. Later, another study suggested a combination of the B-chain with a large excess of the A-chain led to greater yield of natively folded insulin [20]. During the air oxidation step, where the reduced chains are combined to form folded insulin, only a fraction of the total chains is available. The loss of insulin chains in this step is mainly due to their partial insolubility and aggregation, and incorrect disulfide bond arrangement during the initial steps of the protein folding [62, 63]. Moreover, it has been previously indicated that in solution, B-chain has a strong tendency to form soluble aggregates of approximately 40 kDa and its aggregation is also pH dependent [37]. All these facts could explain the very low yield of insulin chain combination in the absence of an appropriate chaperone (**Fig 7A**). The insulin chain combination has been typically applied at pH values between 9.5 and 11 and in the present study it was undertaken in 0.1 M glycine at pH 10.6. The basic pH results in deprotonation of sulfhydryl groups on cysteines (thiolate moieties) and enhances the yield by limiting aggregation of the reduced B-chains. However, due to hydrophobic interactions, partial aggregation of the B-chains occurs at basic pH. All of these challenges reduce the efficiency of insulin folding during the chain combination step [20, 39]. To overcome these problems, Tang et al. used protein disulfide isomerase (PDI) and obtained significantly higher yield compared with the cross-linking of the chains in the absence of this enzyme [62]. PDI has both chaperone and catalytic activities and its chaperone domain prevents aggregation of the target protein, while its catalytic domain catalyzes the correct disulfide formation. However, Winter et al. showed that upon suppressing the catalytic activity of PDI, the yield of insulin combination was maintained at an appreciable level while inhibition of the chaperone function drastically reduced PDI-assisted folding. These findings suggest that the chaperone function of PDI plays the main role during the chain combination and folding of insulin molecules [64]. Hence, the crucial aspect for high efficiency of the chain combination is to select an appropriate chaperone that prevents aggregation of the insulin chains. There are many reports on αB-Cry and lens α-Cry preventing insulin aggregation under reducing conditions [48, 65].

αB-Cry is an ATP-independent molecular chaperone that has little ability to fold target proteins but it stabilizes unfolding target proteins to prevent their aggregation. Therefore, we used αB-Cry as both fusion partner to prepare a suitable construct for the individual expression of human insulin A- and B-chain and later a chaperone to ensure correct folding during the chain combination step (**Fig 13**). Fortunately, the chaperone activity of αB-Cry is still efficient at high pH values [66]. Indeed, αB-Cry significantly increased the yield of insulin chain combination and folding at basic pH (**Fig 7B**). The chaperone also stabilized the native insulin molecules against chemical stress during the subsequent purification step (**Figs 10 and 11**) [42]. As indicated in **Fig 7B**, after 48 h, the yield of natively folded insulin molecules increased from 10.2% in the absence of αB-Cry to 26.7% in its presence. The residual fractions belonged to the isolated A- and B-chain and incorrectly folding intermediates. A highly pure sample of natively folded human recombinant insulin was achieved with a suitable phenyl sepharose column and elution at 250 mM ammonium sulfate. After purification, we compared our insulin with the standard insulin sample according to their structure and biological activity. The results of RP-HPLC analysis, structural assessment by fluorescence, CD and NIR, aggregation and oligomerization studies, as well as *in vivo* activity assessments, all showed that our recombinant insulin exhibited substantial similarity to standard insulin.

Overall, we designed a streamlined insulin chain expression and purification protocol in which all individual steps are straightforward with high yield. Additionally, this study provided a simple and efficient route to express and purify insulin peptide chains that are difficult or expensive to produce by chemical synthesis or by ordinary recombinant methods. Our results also suggest αB-Cry as a remarkable candidate not only to increase the efficacy of insulin folding during the chain combination step but also as a possible interesting excipient in the formulation of this therapeutic hormone.

## Supporting information

S1 Fig RP-HPLC analysis of human αB-Cry. The retention time of αB-Cry used for the chain combination experiment was determined using C18 reverse phase column. A 20 μL of αB-Cry (2 mg/m) in the refolding buffer was subjected to the column and analyzed by Knauer HPLC system. The absorbance signals were recorded at 214 nm using DAD 2.1 UV-Visible detector (Knauer, Germany).
(TIF)
