# Supplementary figures and images for: Human αB-crystallin as fusion protein and molecular chaperone increases the expression and folding efficiency of recombinant insulin
Source: PLoS One. 2018 Oct 19;13(10):e0206169. doi: 10.1371/journal.pone.0206169 (PMC6195290; doi:10.1371/journal.pone.0206169)

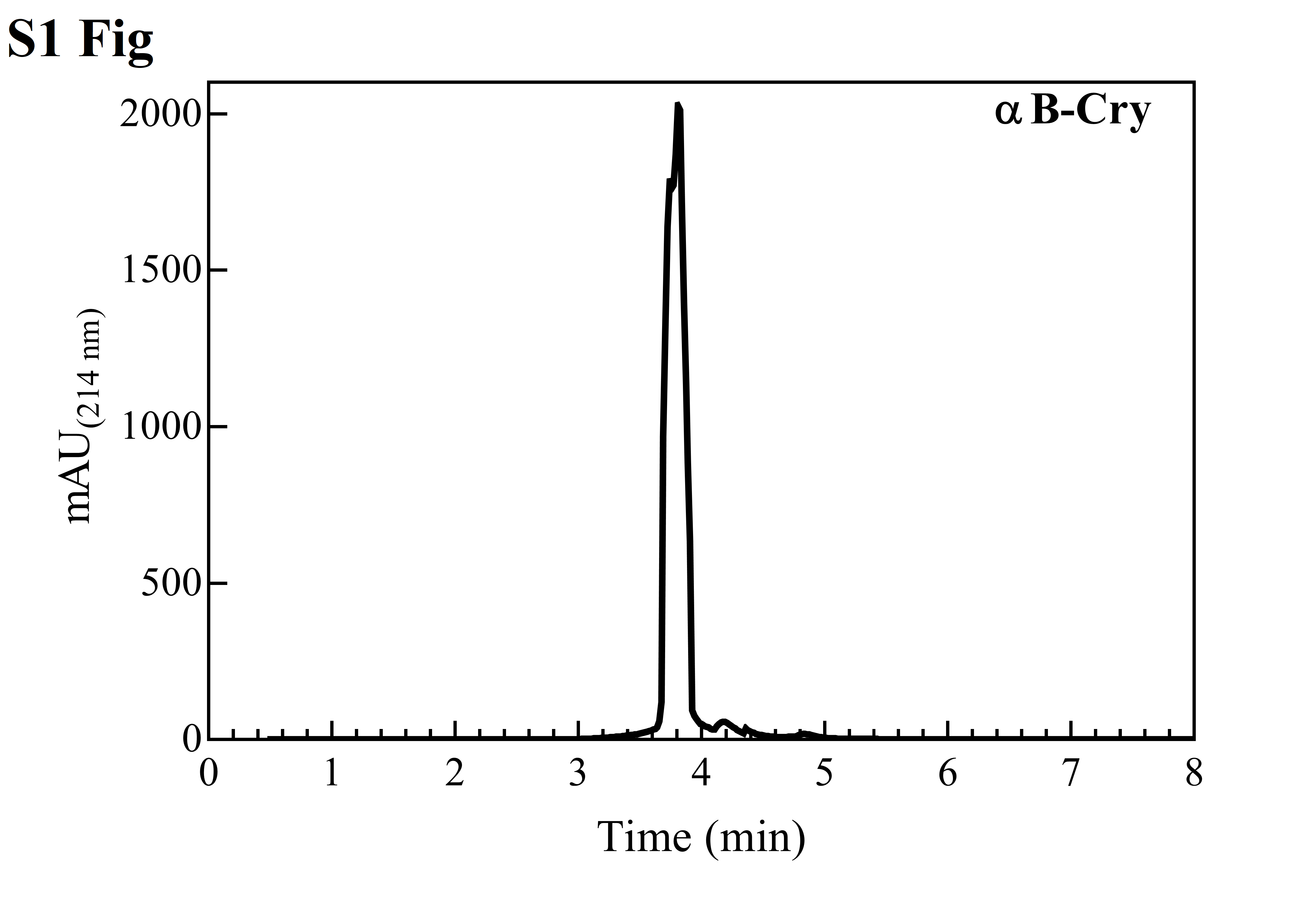

Supplement: S1 Fig — The retention time of αB-Cry used for the chain combination experiment was determined using C18 reverse phase column. A 20 μL of αB-Cry (2 mg/m) in the refolding buffer was subjected to the column and analyzed by Knauer HPLC system. The absorbance signals were recorded at 214 nm using DAD 2.1 UV-Visible detector (Knauer, Germany). (TIF) [file pone.0206169.s001.tif]
